# Identification of Novel Native Autoantigens in Rheumatoid Arthritis

**DOI:** 10.3390/biomedicines8060141

**Published:** 2020-05-29

**Authors:** Thomas B. G. Poulsen, Dres Damgaard, Malene Møller Jørgensen, Ladislav Senolt, Jonathan M. Blackburn, Claus H. Nielsen, Allan Stensballe

**Affiliations:** 1 Department of Health Science and Technology, Aalborg University, 9220 Aalborg, Denmark; 2 Sino-Danish Center for Education and Research, University of Chinese Academy of Sciences, 380 Huaibeizhuang, Huairou district, Beijing 100049, China; 3 Institute for Inflammation Research, Center for Rheumatology and Spine Diseases, Copenhagen University Hospital Rigshospitalet, 2100 Copenhagen, Denmark; dres.damgaard@regionh.dk (D.D.); claus.henrik.nielsen@regionh.dk (C.H.N.); 4 Department of Clinical Immunology, Aalborg University Hospital, 9000 Aalborg, Denmark; maljoe@rn.dk; 5 Department of Clinical Medicine, Aalborg University, 9000 Aalborg, Denmark; 6 Institute of Rheumatology and Department of Rheumatology, 1st Faculty of Medicine, Charles University, 121 08 Prague, Czech Republic; senolt@revma.cz; 7 Department of Integrative Biomedical Sciences & Institute of Infectious Disease and Molecular Medicine, University of Cape Town, Cape Town 7700, South Africa; j.blackburn@sengenics.com; 8 Sengenics Corporation Pte Ltd., Singapore 409051, Singapore

**Keywords:** protein array, rheumatoid arthritis, autoantibodies, native autoantigens, immunome, microarray

## Abstract

The majority of patients diagnosed with rheumatoid arthritis (RA) have developed autoantibodies against neoepitopes in proteins that have undergone post-translational modification, e.g., citrullination or carbamylation. There is growing evidence of their molecular relevance and their potential utility to improve diagnosis, patient stratification, and prognosis for precision medicine. Autoantibodies reacting to native proteins may also have a role in RA pathogenesis, however, their reactivity patterns remain much less studied. We hypothesized that a high-density protein array technology could shed light onto the normal and disease-related autoantibodies produced in healthy and RA patient subgroups. In an exploratory study, we investigated the global reactivity of autoantibodies in plasma pools from 15 anti-cyclic citrullinated peptide (CCP)-positive and 10 anti-CCP-negative RA patients and 10 healthy donors against more than 1600 native and unmodified human proteins using a high-density protein array. A total of 102 proteins recognized by IgG autoantibodies were identified, hereof 86 were recognized by antibodies from CCP-positive RA patients and 76 from anti-CCP-negative RA patients, but not by antibodies from healthy donors. Twenty-four of the identified autoantigens have previously been identified in synovial fluid. Multiple human proteins in their native conformation are recognized by autoantibodies from anti-CCP-positive as well as anti-CCP-negative RA patients.

## 1. Introduction

Rheumatoid arthritis (RA) is the most common systemic autoimmune disease characterized by synovial inflammation that can cause joint damage, disability, and may be associated with increased prevalence of systemic manifestations, including, e.g., cardiovascular comorbidities [1]. The identification of novel autoantibodies and autoantibody reactivity patterns may improve our understanding of pathogenesis and enable personalized stratification of patients into subsets that may benefit from different treatment modalities [2,3,4,5,6].

Before disease phenotype onset, a pre-RA phase lasting months to years, may be characterized by the presence of circulating autoantibodies in biofluids, increasing concentration and range of inflammatory cytokines and chemokines, and altered metabolism [7]. Around 2/3 of RA patients produce anti-citrullinated protein antibodies (ACPAs), measured in vitro as anti-CCP antibodies, which are utilized as key clinical diagnostic biomarkers in RA [8,9]. The etiopathogenesis and clinical course of this patient subset differ from those of ACPA-negative RA patients [10]. However, both ACPA-positive and ACPA-negative patients show antibody reactivity against native proteins [11,12,13,14].

During the last five years, more than 20 native proteins have been found to be recognized by autoantibodies from RA patients [15,16]. In a recent study, antibody reactivity against native hnRNP A2/B1 (RA33) was found in synovial fluid from RA patients, primarily those with early RA, whereas antibodies against citrullinated RA33 were primarily found in patients with a longer disease duration and more erosive disease [17]. This challenges the common view that the loss of tolerance depends on citrullination [18,19]. Furthermore, a recent study by Zheng et al. demonstrated the binding of IgG antibodies to native peptides in anti-CCP- and rheumatoid factor (RF)-positive RA, even to peptides that do not contain arginine or lysine and therefore are not likely to be recognized by cross-reacting antibodies against citrulline or homocitrulline [20]. This underlines the importance of investigating antibody reactivity against native proteins being autoantigen targets in RA. Such efforts are supported by novel technologies such as multiplex platforms and high-density protein arrays providing a high sensitivity, high reproducibility and high dynamic range [21,22,23].

Here, we investigate the autoantibody reactivity against native (non-post translational modified) full-length proteins in anti-CCP-positive and anti-CCP-negative RA patients using a microarray containing more than 1600+ immune-related native human proteins to explore the complement of autoantibodies against native proteins in humans.

## 2. Experimental Section

### 2.1. Collection of Plasma Samples

Plasma was isolated from 15 anti-CCP-positive RA patients with median anti-CCP levels of 274 mU (age: 52.8 ± 15.6 (27–84), sex: 10 female, 2 male, and 3 unknown, DAS28-ESR: 5.1 ± 1.4, DAS28-CRP: 4.7 ± 1.2, CRP (mg/L): 29.4 ± 31.8, anti-CCP: 531.2 ± 526.8, RF IgM: 91.7 ± 126.3, 5 in csDMARDs treatment and 13 in bDMARDs treatment), 10 anti-CCP-negative patients with median anti-CCP levels of 7.9 mU (age: 53.8 ± 17.1 (34–81), sex: 5 female and 5 male, DAS28-ESR: 3.7 ± 0.8, DAS28-CRP: 4 ± 1.2, CRP (mg/L): 12.6 ± 8.9, anti-CCP: 6.1 ± 6.4, RF IgM: 21.6 ± 19.7, 1 in csDMARDs treatment and 7 in bDMARDs treatment), and 10 anti-CCP-negative healthy donors. Plasma from each group were pooled prior to analysis. The RA patients fulfilled the American College of Rheumatology and European League Against Rheumatism criteria for RA diagnosis [1], and the ethics committee of the Institute of Rheumatology in Prague, Czech Republic, approved the use of patient samples after informed patient consent (26 June 2012, No. 3294/2012). Plasma samples from donors attending the Blood Bank at Copenhagen University Hospital, Rigshospitalet, were used as controls. All healthy blood donors were anonymous to the investigators.

### 2.2. Protein Microarray Treatment

After the removal of storage buffer, Immunome™ protein microarrays containing 1600+ immune-related human proteins in a native configuration spotted in quadruplicates (Sengenics, Singapore) were washed twice in cold phosphate-buffered saline (PBS) at 4 °C using a Pap jar (Fischer scientific, Pittsburgh, PA, USA) and were wiped at the edges between each wash. Subsequently, the microarrays were added to the quadriperm chamber (Greiner BioOne, Kremsmünster, Austria) containing the reaction buffer (1 mM Dithiothreitol, 10 mM CaCl2, 100 mM Tris-HCl) and incubated at 37 °C for 3 h while shaking at 50 rpm. Slides were removed, dried, and washed in cold serum albumin buffer (SAB) (0.1% Triton X-100, 0.1% bovine serum albumin in PBS) in a slide tube, inverted 4 times and shaken for 5 min at 50 RPM on a shaker (IKA, Germany, Königswinter). This step was repeated once with new SAB buffer. Then, 30 µL pooled plasma from patients or healthy controls (diluted 1:200 in SAB buffer) was added, and the slides were incubated on a shaker for 2 h at ambient temperature (20 °C) at 50 RPM. The slides were removed from the chamber, dried, and washed in a slide tube containing SAB buffer by inverting them 4 times and shaking them for 20 min on a shaker at 50 RPM at ambient temperature. This was repeated twice and followed by incubation in a quadriperm chamber containing SAB buffer and Cy3 conjugated (GE Healthcare, Chicago, Ill, USA) polyclonal rabbit anti-human IgG (Dako, Santa Clara, CA, USA), diluted 1:1000 (*v*/*v*) and covered in tinfoil for 2 h at 50 RPM on a shaker at ambient temperature. The slides were then removed and washed with 30 mL SAB buffer on a shaker for 5 min at 50 RPM at ambient temperature, which was repeated once and followed by washing in ultrapure water. Finally, the slides were centrifuged at 240 G for 5 min in a new slide tube in order to dry. Slides were scanned on a high-resolution slide reader (Innoscan 710AL, Innopsys, Carbonne, France) using the Mapix software (Ver. 8.2.2, Innopsys). The chosen scan settings were a 532 nm laser with a low laser power (5V), PMT gain at 60%, 5 µm resolution, and a scan speed of 35 px/sec. The images were exported as TIF format to ensure high-quality pictures.

### 2.3. Extraction of Quantitative Expression Data

Scanned images of the microarrays were imported to Spotxel (SICASYS, v1.7.6, Heidelberg, Germany). The GAL file specific for the Immunome™ microarray automatically aligned the protein spot grid using Spotxel. This was followed by quantification of the array using the Flex-Spot option ensuring that every protein spot was included, even if the spot differed from the specific location specified by the GAL file coordinates. Following quantification, we examined the boundaries of the protein spots to ensure that they represented the protein spots, and that any artifact present on the microarray was not included in the analysis. The raw data were imported to R (Ver 1.1.456, R Core Team), and data were processed using an in-house developed R analysis. Raw data can be found in Table S1.

### 2.4. Data Analysis Pipeline

**Figure 1:**
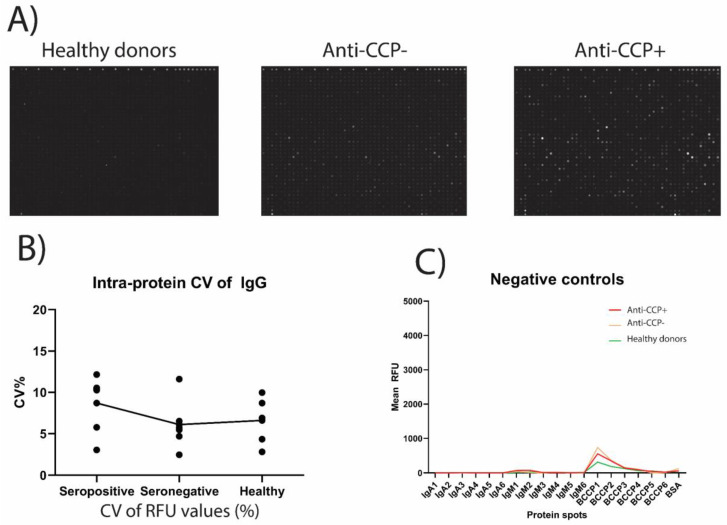
Immunome microarray images and corresponding quality control plots. Pooled plasma from anti-cyclic citrullinated peptide (CCP)-negative healthy donors (*n* = 10), anti-CCP-negative rheumatoid arthritis (RA) patients (*n* = 10), or anti-CCP-positive RA patients (*n* = 15) were added to Immunome™ microarrays containing human proteins in their native configuration spotted in quadruplicates on each microarray. The binding of IgG autoantibodies was subsequently detected using fluorescence-labelled goat anti-human IgG antibodies and scanned on an Innoscan 710AL slide reader. (**A**) Scanned microarrays containing one of the four replicates on each microarray. Fluorescent spots indicate positive binding of autoantibodies from plasma. (**B**) Intra-protein coefficient of variation (CV) for six IgG-positive control spots on the microarray demonstrating bound anti-human IgG antibody. (**C**) Negative control spots for secondary IgG antibody alone showing no binding to IgA or IgM replicates or the marker of correct protein-folding, biotin-carboxyl carrier protein (BCCP), or bovine serum albumin (BSA).

Scanned Raw intensity values were used to normalize the data using a combination of quantile- and intensity-based normalization as described [24]. Normalized expression values were used to calculate a Z-score, coefficient of variation (CV), and Chebyshev inequality precision (CI-P) for each protein. Cut-off values were Z-score > 2, Intra-protein CV < 15, and CI-P < 0.05. These restrictions were applied in order to filter low intensities away, ensuring high reproducibility between quadruplicates, and to make sure the intensities did not belong to the negative control distribution. Quality control for the secondary antibody used included positive and negative immunoglobulin controls which confirmed the high-quality labeling efficiency and spot detection of the secondary antibody. Spots included IgG for a positive control and IgA and IgM for negative controls (Figure 1). Following data filtering, we applied a two-sample t-test with a Benjamini–Hochberg false discovery rate to identify statistically significant results. Fold changes for the filtered results were calculated between each patient/donor group.

## 3. Results

Pools of plasma from 15 anti-CCP-positive RA patients, 10 anti-CCP-negative RA patients, and 10 anti-CCP-negative healthy donors were each applied to the commercially available Immunome™ Discovery microarray consisting of more than 1600+ different human proteins from different protein families including kinases, signaling molecules, cytokines, interleukins, chemokines, and cancer antigens [25,26] (Figure 1 and Figure S1).

The average intensities measured across all three conditions were all relatively low (< 2500 relative fluorescence units (RFU)), indicating low antibody activity against most proteins. The top 10% highest intensities associated with the anti-CCP-positive plasma reached an average intensity of 12,500 RFU, while less than half and around 1/10 of this intensity were observed after incubation with plasma from the anti-CCP-negative patients and healthy donors, respectively (Figure S2).

### 3.1. Identification of Native Autoantigens

**Figure 2:**
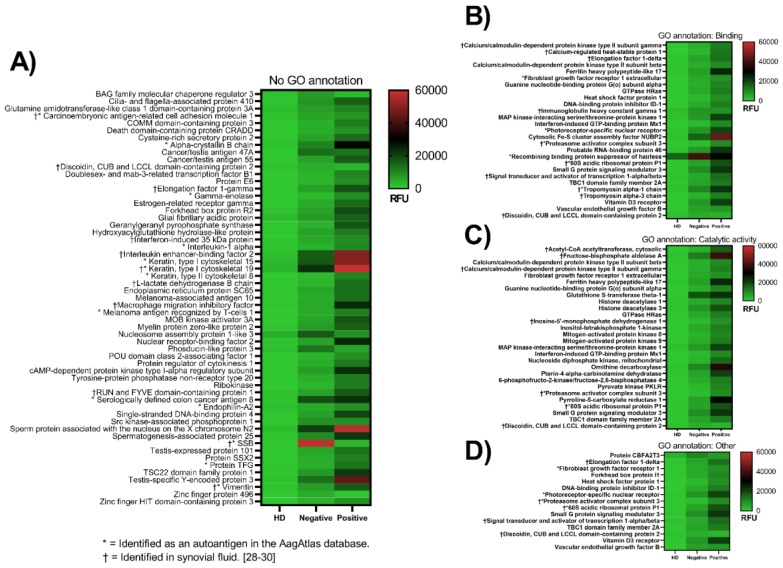
Native proteins recognized by antibodies from anti-CCP-positive and anti-CCP-negative RA patients. Pooled plasma from healthy donors (HD, *n* = 10), anti-CCP-positive RA patients (positive, *n* = 15), and anti-CCP-negative RA patients (negative, *n* = 10) were tested for antibody reactivity against 1600+ immune-related native human proteins using the Immunome™ protein microarray. Shown are four different heatmaps categorized according to the antigens’ gene ontology annotation. The values represented are the antibody reactivities expressed as relative fluorescence units (RFU). (**A**) Proteins that could not be assigned a molecular function GO annotation, (**B**) proteins involved in binding, (**C**) proteins involved in catalytic activity, and (**D**) the GO annotation other includes molecular function regulator and transducer activity, structural molecule activity, and transcription regulator activity. Proteins included in the figure meet the following criteria: Fold change > 2, z-score > 2, intra-protein percentage coefficient of variation (%CV) < 15, Chebyshev inequality precision (CI-P) < 0.05, and *p*-value < 0.05.

We identified 102 native proteins that were recognized by antibodies in the anti-CCP-positive plasma pool and/or the anti-CCP-negative plasma pool, with fold changes ranging from two to more than 100 compared with the plasma pool from healthy donors (Figure 2 and Table S2 and Table S3). Of these, 23 have previously been identified as autoantigens according to the human autoantigen database AagAtlas [27]. Gene ontology (GO) annotation analysis of the identified proteins’ molecular functions categorized several of them as involved in either the binding or catalytic activity (Figure 2B,C). The remaining proteins could either not be assigned a GO annotation (Figure 2A) or were involved in molecular function regulation or transducer activity, or structural molecular activity or transcription regulator activity (Figure 2D).

Of the 102 autoantigens identified in this study, 86 and 76 were recognized by antibodies from anti-CCP-positive and anti-CCP-negative RA patients, respectively, with an overlap of 61 proteins. Among the overlapping proteins, 12 were listed as known autoantigens in the AagAtlas dataset: alpha-crystallin B chain, fibroblast growth factor receptor 1 (FGFR1), histone deacetylase 1 and 3, keratin type I cytoskeletal 15 and 19 (KRT15 and KRT19), mitogen-activated protein kinase 9, melanoma antigen recognized by T-cells 1, photoreceptor-specific nuclear receptor, serologically defined colon cancer antigen 8, endophilin-A2, and vimentin.

One protein, FAS-associated factor 1, was recognized by antibodies from healthy donor plasma to a moderately higher degree than by antibodies from anti-CCP-positive RA plasma (2300 RFU vs. 1000 RFU). The antibody binding was generally higher in the presence of anti-CCP-positive plasma than in presence of anti-CCP-negative plasma or healthy donor plasma (Figure 1 and Figure 2).

The biggest fold differences in antibody binding between anti-CCP-positive RA patients and healthy donors were observed for spots containing the calcium-regulated heat-stable protein 1 (35-fold compared with healthy donor plasma), testis-specific Y-encoded protein 3 (28-fold), and Cytosolic Fe-S cluster assembly factor NUBP2 (28-fold) (Figure 2 and Table S2). The biggest differences between anti-CCP-negative and healthy donors were found for SSB (121-fold), cancer/testis antigen 47A (23-fold), and glutathione S-transferase theta-1 (18-fold). Of these, only SSB has previously been identified as an autoantigen according to the human autoantigen database AagAtlas.

### 3.2. Differences in Antibody Reactivity between Anti-CCP-Positive and Anti-CCP-Negative Plasma

For 71 proteins, significantly stronger reactions were observed in the presence of anti-CCP-positive than in the presence of anti-CCP-negative RA patient plasma (Figure 2, Table S2 and Table S3). The opposite was true for four proteins (SSB, zinc finger protein 496, BAG family molecular chaperone regulator 3, and phosducin-like 3). The differences were generally less than 5-fold. SSB showed 19 times higher antibody binding after incubation with anti-CCP-negative plasma than with anti-CCP-positive plasma, while the binding to zinc finger protein 496, BAG family molecular chaperone regulator 3, and phosducin-like 3 was up to 4-fold higher.

A total of 15 out of the 75 mentioned proteins showing differential antibody binding (71 highest in the presence of anti-CCP-positive plasma, and 4 highest in the presence of anti-CCP-negative plasma) have previously been identified as a disease-related autoantigen according to the AagAtlas database.

### 3.3. Antigens Identified in Synovial Fluid

In the search for potentially pathogenic RA autoantigens, we investigated which of the 102 identified autoantigens are present in synovial fluid proteome, essentially being a plasma filtrate [28]. We found 24 proteins that have previously been shown to be present in synovial fluid (Table 1). Extracellular proteins included vimentin, acetyl-CoA acetyltransferase, fructose-bisphosphate aldolase A, KRT19, L-lactate dehydrogenase, macrophage migration inhibitory factor, transketolase, tropomyosin, carcinoembryonic antigen-related cell adhesion molecule 1, elongation factor 1-gamma, and inosine-5′-monophosphate dehydrogenase 1. Especially KRT19 showed high binding intensity after incubation with plasma from anti-CCP-positive RA patients compared with plasma from healthy donors (17-fold). Of note, IgG antibodies against immunoglobulin heavy constant gamma 1 (IgG rheumatoid factor) were also identified with a 4- and 3-fold increase after incubation with anti-CCP-positive plasma and anti-CCP-negative plasma compared with healthy donor plasma.

## 4. Discussion

While antibody binding to post-translationally modified antigens in RA has been studied intensively, few studies have investigated the binding of the patients’ antibodies to native autoantigens [11,12,13,14,15,16,18]. To our knowledge, this study is the first to use high-density protein arrays for native autoantigen profiling in RA.

The existence of polyreactive, naturally occurring autoantibodies, mainly of IgM but also of other Ig isotypes, has been known for decades [32]. However, in this study, we focused on IgG autoantibodies. We identified 102 native proteins as potential autoantigens. Antibodies from anti-CCP-positive patients recognized 86 of these proteins, at least 18 of which have previously been identified as autoantigens [27]. Of these, both native and citrullinated vimentin is a known target for autoantibodies in RA, while interleukin-1 alpha and KRT8 are recognized in their native state [33,34,35,36]. Autoantibodies against native KRT15 were found in patients suffering from RA with secondary vasculitis [37]. The remaining 14 autoantigens have been identified in diseases such as multiple sclerosis, asthma, and cancer [38,39,40]. Antibodies in anti-CCP-negative plasma recognized 76 proteins, 17 of which have previously been shown to be recognized by autoantibodies. Of these, three proteins have been targets for autoantibodies in RA including the aforementioned KRT15 and vimentin, but also SSB, which is recognized in its native state [41,42].

This study identifies 12 proteins recognized by antibodies contained in both anti-CCP-positive and anti-CCP-negative RA plasma that are also listed as autoantigens in the AagAtlas database, but not necessarily to RA. This includes vimentin, cytokeratins KRT15 and KRT19, and FGFR1.

Vimentin is an intracellular protein that is also known to be secreted from activated macrophages and expressed on the cellular surface of monocytes [43,44]. Post-translationally modified vimentin, e.g., phosphorylation, ubiquitination, carbamylation, and citrullination, has been extensively studied and shown to be involved in several diseases including RA, systemic lupus erythematosus, and ankylosing spondylitis [45,46,47,48,49,50]. It is a promising diagnostic and prognostic marker in RA with sensitivity and specificity comparable to anti-CCP [51,52,53,54]. The role of native vimentin is much less studied in RA, but it may prove a useful biomarker.

The cytokeratins KRT15 and KRT19 in native form appeared to be targets for autoantibodies in both anti-CCP-positive and anti-CCP-negative RA, and with a 17-fold increase over the background signal defined by donor serum, and native KRT19 stood out as a prominent autoantigen in anti-CCP-positive RA. It has not previously been suggested as an autoantigen in RA, but IgG autoantibodies against KRT19 have been identified in serum from mice exposed to cigarette smoke [55], which is notable given the strong association between smoking and anti-CCP-positive RA [56]. Even though it is possible for intracellular antigens to be bound by antibodies outside the cell, e.g., after leakage from dying cells, extracellular antigens are presumably more likely to form pathogenic complement- and leukocyte-activating immune complexes with autoantibodies than intracellular antigens.

An apparent paradoxical reactivity was observed against FGFR1, a member of the FGFR family which acts as a receptor for specific fibroblast growth factor (FGF) family members that are important homeostatic growth factors in joint tissue and regulate cartilage homeostasis [57,58]. FGF2 binding to FGFR1 leads to angiogenesis which is part of the pathogenesis of RA during pannus formation [59,60]. Another result of FGFR1 activation by FGF2 promoted the catabolism and impeded the anabolism of human articular chondrocytes [61]. Thus, signaling through FGFR1 potentially has a degenerative function in the joint, and blocking antibodies may therefore be protective. It remains to be shown whether the anti-FGFR1 antibodies demonstrated in this study are receptor-blocking or stimulatory; theoretically, a stimulatory effect may be pathogenic.

SSB was identified as an autoantigen in anti-CCP-negative RA, specifically. The production of anti-SSB antibodies is a hallmark of Sjögren’s syndrome, but can also be found in systemic lupus erythematosus [62] and RA patients [41,63]. Since we used pooled plasma in this study, one or a few patients in the anti-CCP-negative cohort may be responsible for this reactivity. The overlap between RA and lupus, referred to as “rhupus”, is well-described [64]. However, none of the patients included in the cohort were diagnosed with systemic lupus erythematosus.

The finding of IgG autoantibodies against IgG1, i.e. IgG rheumatoid factor (IgG RF), in pools of both anti-CCP-positive and anti-CCP-negative plasma was expected. Although there is overlap between ACPA and RF production, it is well-recognized that RF is also produced by subsets of ACPA-negative patients [65,66].

Rheumatoid arthritis is characterized by synovial inflammation. The synovial membrane is in direct contact with the synovial fluid within the joints making the synovial fluid interesting in RA due to being present at the site of inflammation. Although this study used plasma to investigate the presence of autoantibodies in RA patients, synovial fluid is an ultrafiltrate from plasma. Thus, the proteomes share many similarities, however, plasma lack the proteins secreted from the surrounding tissue in the synovium [28,67,68,69]. For these autoantigens to be pathologically relevant, we suspect they need to be at the site of disease in the joints. Therefore, it is interesting whether the identified autoantigens previously have been identified in synovial fluid as well. Following this logic, we identify 24 potential pathologically relevant autoantigens. The pathological events leading to inflammation in RA may be due to the generation of immune complexes that drive the local production of antibodies against native proteins within the joint, just like the suggested action of ACPAs’ presence in the synovial joint [70]. However, whether the identified autoantigens present in synovial fluid are indeed pathologically relevant is not known.

A limitation to our study is the use of pooled plasma samples, which do not allow a demonstration of differences between individual patients or subgroups of RA patients. Patients with RA often present with different symptoms, signs, and response to treatment [71,72,73,74]. Even patients presenting with the same clinical symptoms may differ with respect to cell infiltration, cytokine involvement, and gene activation and expression [75,76,77,78,79]. The design of custom arrays specifically for RA including both known autoantigens, native as well as post-translationally modified autoantigens, and novel promising targets would enable a thorough stratification of RA patients based on their antibody fingerprint, benefitting personalized diagnosis and treatment.

## 5. Conclusions

This study explored the autoantigen repertoire in normal and RA patients. We identify several native autoantigens recognized by autoantibodies in both anti-CCP-positive and anti-CCP-negative RA including vimentin, FGFR1, KRT15, and KRT19. This demonstrates that autoimmunity in RA is not restricted to post-translationally modified epitopes, and that reactivity against native proteins is higher in anti-CCP-positive RA plasma than in anti-CCP-negative RA plasma.

## Figures and Tables

**Table 1:** Synovial fluid proteins identified as autoantigens in this study.*.

Gene Name	Protein Name	Localization †
IGHG1 (IgG1)	Immunoglobulin heavy constant gamma 1	Extracellular/cell associated
ACAT2	Acetyl-CoA acetyltransferase	Intracellular and extracellular
ALDOA	Fructose-bisphosphate aldolase A	Intracellular and extracellular
CEACAM1	Carcinoembryonic antigen-related cell adhesion molecule 1	Intracellular and extracellular
EEF1G	Elongation factor 1-gamma	Intracellular and extracellular
IMPDH1	Inosine-5’-monophosphate dehydrogenase 1	Intracellular and extracellular
KRT19	Keratin 19	Intracellular and extracellular
LDHB	L-lactate dehydrogenase B chain	Intracellular and extracellular
MIF	Macrophage migration inhibitory factor	Intracellular and extracellular
TKT	Transketolase	Intracellular and extracellular
TPM3	Tropomyosin alpha-3 chain	Intracellular and extracellular
VIM	Vimentin	Intracellular and extracellular
CAMK2G	Calcium/calmodulin-dependent protein kinase type II subunit gamma	Intracellular
CARHSP1	Calcium-regulated heat-stable protein 1	Intracellular
EEF1D	Elongation factor 1-delta	Intracellular
IFI35	Interferon-induced 35 kDa protein	Intracellular
ILF2	Interleukin enhancer-binding factor 2	Intracellular
PRKAR1A	cAMP-dependent protein kinase type I-alpha regulatory subunit	Intracellular
PSME3	Proteasome activator complex subunit 3	Intracellular
RPLP1	60S acidic ribosomal protein P1	Intracellular
RUFY1	RUN and FYVE domain-containing protein 1	Intracellular
SSB	Lupus La protein / SSB	Intracellular
STAT1	Signal transducer and activator of transcription 1-alpha/beta	Intracellular
TPM1	Tropomyosin alpha-1 chain	Intracellular

* Identified in synovial fluid in the following studies [29,30,31]. † According to uniprot.org.

## Supplementary Materials

Supplementary materials can be found at https://www.mdpi.com/2227-9059/8/6/141/s1. Figure S1: Scanned Immunome microarray, Figure S2: General intensity of rheumatoid arthritis and healthy donor plasma, Table S1: Excel document containing all information about protein spots on the microarray and their corresponding relative fluorescence unit intensity from plasma antibody binding from healthy donors and anti-CCP-positive and anti-CCP-negative RA patients, Table S2: Fold changes for all filtered positive hits between all 3 conditions, Table S3: Unique targeted antigens in one of the two rheumatoid arthritis conditions.
