# Supplementary material for: Identification of Novel Native Autoantigens in Rheumatoid Arthritis
Source: Biomedicines. 2020 May 29;8(6):141. doi: 10.3390/biomedicines8060141 (PMC7345460; doi:10.3390/biomedicines8060141)
Supplement: Supplementary file 1 [file biomedicines-08-00141-s001.zip › biomedicines-817876-supl.pdf]

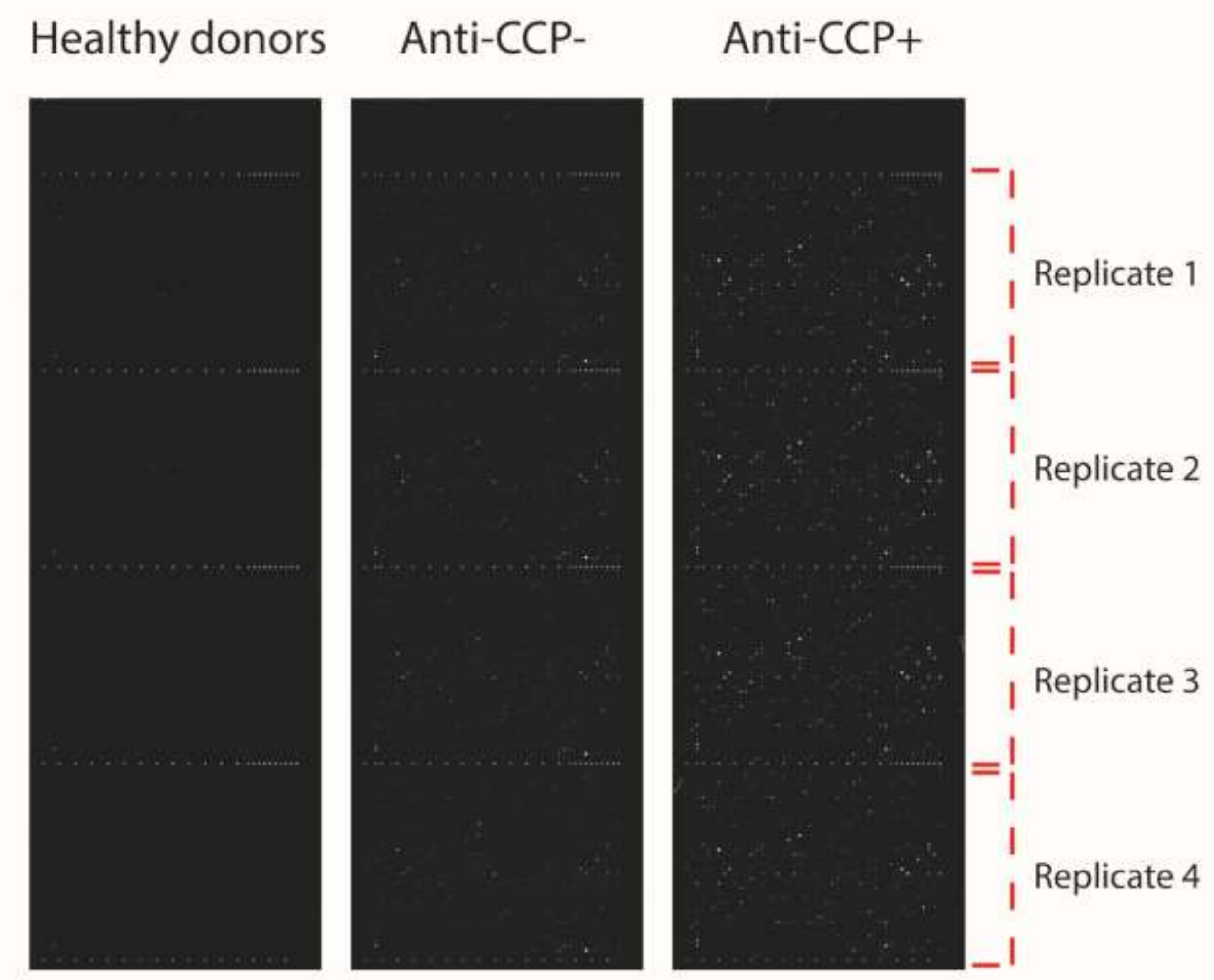

Figure S1. Scanned Immunome microarray. The microarrays including the four on-slide replicates containing a total of more than 1600 proteins in quadruplicates.

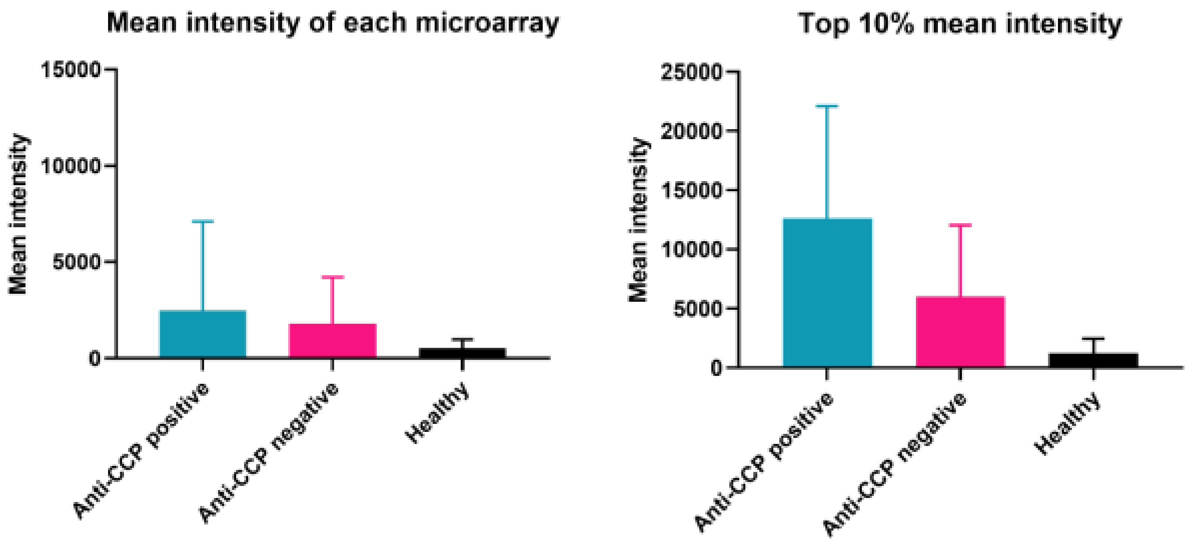

Figure S2. General intensity of rheumatoid arthritis and healthy donor plasma. The average intensity of all proteins included on the microarrays after the addition of anti-CCP positive or anti-CCP negative rheumatoid arthritis plasma or healthy donor plasma. It also demonstrates the average intensity of the top 10% most intense targets on each slide, demonstrating the general intensity across each population of patients.

Table S1. Native proteins recognized by antibodies in plasma from anti-CCP positive or anti-CCP negative RA patients*.

Protein	Gene	Anti-CCP positive plasma vs healthy donor plasma (fold-change)	Anti-CCP negative plasma vs healthy donor plasma (fold-change)	Unique identified in one of the two RA groups: Anti-CCP positive vs Anti-CCP negative plasma (fold-change)	Identified in the human autoantigen database (AagAtlas)[a]
Acetyl-CoA acetyltransferase, cytosolic	ACAT2	20		4	
Fructose-bisphosphate aldolase A	ALDOA	26	9		
BAG family molecular chaperone regulator 3	BAG3		5	0.4	
Cilia- and flagella-associated protein 410	C21orf2	14	7		
Glutamine amidotransferase-like class 1 domain-containing protein 3A	C21orf33	5	3		
Calcium/calmodulin-dependent protein kinase type II subunit beta	CAMK2B	9	4		
Calcium/calmodulin-dependent protein kinase type II subunit gamma	CAMK2G	13	5		
Calcium-regulated heat-stable protein 1	CARHSP1	35		4	
Protein CBFA2T3	CBFA2T3		3		
Carcinoembryonic antigen-related cell adhesion molecule 1	CEACAM1		3		Yes.
COMM domain-containing protein 3	COMMD3	17		3	
Death domain-containing protein CRADD	CRADD	11			
Cysteine-rich secretory protein 2	CRISP2	15	7		
Alpha-crystallin B chain	CRYAB	20	7		Yes.

Cancer/testis antigen 47A	CT47A1	19	23		
Cancer/testis antigen 55	CXorf48	17	6		
Discoidin, CUB and LCCL domain-containing protein 2	DCBLD2		3		
Doublesex- and mab-3-related transcription factor B1	DMRTB1	15		3	
Protein E6	E6	14		3	
Elongation factor 1-delta	EEF1D	12	5		
Elongation factor 1-gamma	EEF1G	18	6		
Gamma-enolase	ENO2	15		3	Yes.
Estrogen-related receptor gamma	ESRRG	22	8		
Fibroblast growth factor receptor 1 extracellular	FGFR1_ext	12	7		Yes.
Forkhead box protein I1	FOXI1	11	5		
Forkhead box protein R2	FOXR2	15	5		
Ferritin heavy polypeptide-like 17	FTHL17		5	4	
Glial fibrillary acidic protein	GFAP	6	3		
Geranylgeranyl pyrophosphate synthase	GGPS1	20		3	
Guanine nucleotide-binding protein G(o) subunit alpha	GNAO1	12		2	
Glutathione S-transferase theta-1	GSTT1		18		
Hydroxyacylglutathione hydrolase-like protein	HAGHL	12	6		
Histone deacetylase 1	HDAC1	7	3		Yes.
Histone deacetylase 3	HDAC3	14	7		Yes.
GTPase HRas	HRAS	12	5		
Heat shock factor protein 1	HSF1	16		3	

DNA-binding protein inhibitor ID-1	ID1	14	6		
Interferon-induced 35 kDa protein	IFI35	11	6		
Immunoglobulin heavy constant gamma 1	IGHG1	4	3		
Interleukin-1 alpha	IL1A	8		3	Yes.
Interleukin enhancer-binding factor 2	ILF2	26	9		
Inosine-5'-monophosphate dehydrogenase 1	IMPDH1	14		3	
Inositol-tetrakisphosphate 1-kinase	ITPK1	14	7		
Keratin, type I cytoskeletal 15	KRT15	17	6		Yes.
Keratin, type I cytoskeletal 19	KRT19	17	7		Yes.
Keratin, type II cytoskeletal 8	KRT8	21		4	Yes.
L-lactate dehydrogenase B chain	LDHB	16		4	
Endoplasmic reticulum protein SC65	LEPREL4	16	7		
Melanoma-associated antigen 10	MAGEA10	17	6		
Mitogen-activated protein kinase 8	MAPK8_tv1	14		3	
Mitogen-activated protein kinase 9	MAPK9	13	5		Yes.
Macrophage migration inhibitory factor	MIF	24	5		
MAP kinase-interacting serine/threonine-protein kinase 1	MKNK1	11	4		
Melanoma antigen recognized by T-cells 1	MLANA	12	5		Yes.

MOB kinase activator 3A	MOBKL2A	21	7		
Myelin protein zero-like protein 2	MPZL2	16	7		
Interferon-induced GTP-binding protein Mx1	MX1	15		4	
Nucleosome assembly protein 1-like 3	NAP1L3	3	5		
Nucleoside diphosphate kinase, mitochondrial	NME4	21		4	
Photoreceptor-specific nuclear receptor	NR2E3	19	7		Yes.
Nuclear receptor-binding factor 2	NRBF2		5	4	
Cytosolic Fe-S cluster assembly factor NUBP2	NUBP2	28	10		
Ornithine decarboxylase	ODC1	26	8		
Pterin-4-alpha-carbinolamine dehydratase	PCBD	19	6		
Phosducin-like protein 3	PDCL3		7	0.4	
6-phosphofructo-2-kinase/fructose-2,6-bisphosphatase 4	PFKFB4	15	6		
Pyruvate kinase PKLR	PKLR	11		3	
POU domain class 2-associating factor 1	POU2AF1	12		2	
Protein regulator of cytokinesis 1	PRC1	13	5		
cAMP-dependent protein kinase type I-alpha regulatory subunit	PRKAR1A	24	7		
Proteasome activator complex subunit 3	PSME3	13		4	Yes.
Tyrosine-protein phosphatase non-receptor type 20	PTPN20A	17	7		
Pyrroline-5-carboxylate reductase 1	PYCR1		6	4	

Ribokinase	RBKS	18		4	
Probable RNA-binding protein 46	RBM46	17	6		
Recombining binding protein suppressor of hairless	RBPJ		3		Yes.
60S acidic ribosomal protein P1	RPLP1		7	3	Yes.
RUN and FYVE domain-containing protein 1	RUFY1	12	5		
Serologically defined colon cancer antigen 8	SDCCAG8	16	13		Yes.
Small G protein signaling modulator 3	SGSM3	21	9		
Endophilin-A2	SH3GL1	4	3		Yes.
Signal transducer and activator of transcription 1-alpha/beta	STAT1	10	4		
Single-stranded DNA-binding protein 4	SSBP4	17	6		
Src kinase-associated phosphoprotein 1	SKAP1	12	12		
Sperm protein associated with the nucleus on the X chromosome N2	SPANXN2	22	8		
Spermatogenesis-associated protein 25	SPATA25	19	8		
SSB	SSB		121	0.1	Yes.
TBC1 domain family member 2A	TBC1D2	12	6		
Testis-expressed protein 101	TEX101	16	7		
Protein SSX2	SSX2	15	7		
Protein TFG	TFG	14		3	Yes.
Transketolase	TKT	15		3	Yes.
Tropomyosin alpha-1 chain	TPM1		8		Yes.

Tropomyosin alpha-3 chain	TPM3	11		3	
TSC22 domain family protein 1	TSC22D1	19	7		
Testis-specific Y-encoded protein 3	TSPY3	28	9		
Vitamin D3 receptor	VDR	21	8		
Vascular endothelial growth factor B	VEGFB		10		
Vimentin	VIM	13	5		Yes.
Zinc finger protein 496	ZNF496		10	0.2	
Zinc finger HIT domain-containing protein 3	ZNHIT3	12		2	

Fold changes for all filtered positive hits between all 3 conditions. This table include the fold changes between conditions for all the differences in intensity after the addition of anti-CCP positive or anti-CCP negative rheumatoid arthritis plasma or healthy donor plasma. Proteins identified as human autoantigens in the AagAtlas databse are also highlighted.

Table S2. Native proteins targeted differentially by antibodies in anti-CCP positive and anti-CCP negative plasma. *.

Protein	Gene	Mean relative fluorescence units (RFU), anti-CCP positive plasma	Mean RFU, anti-CCP negative	Fold-change	Identified in the human autoantigen database (AagAtlas) [a]
Acetyl-CoA acetyltransferase, cytosolic	ACAT2	15827	4059	4	
BAG family molecular chaperone regulator 3	BAG3	2996	6787	0.4	
Calcium-regulated heat-stable protein 1	CARHSP1	12402	3126	4	
COMM domain-containing protein 3	COMMD3	11418	4328	3	
Doublesex- and mab-3-related transcription factor B1	DMRTB1	10638	3689	3	
Protein E6	E6	8879	3253	3	
Gamma-enolase	ENO2	9157	2836	3	Yes.
Ferritin heavy polypeptide-like 17	FTHL17	19853	6201	4	
Geranylgeranyl pyrophosphate synthase	GGPS1	14590	4236	3	
Guanine nucleotide-binding protein G(o) subunit alpha	GNAO1	10452	4322	2	
Heat shock factor protein 1	HSF1	8532	3002	3	
Interferon-induced GTP-binding protein Mx1	MX1	9574	2724	4	
Interleukin-1 alpha	IL1A	9810	3825	3	Yes.
Inosine-5'-monophosphate dehydrogenase 1	IMPDH1	8285	2881	3	
Keratin, type II cytoskeletal 8	KRT8	10368	2984	4	Yes.
L-lactate dehydrogenase B chain	LDHB	12630	3564	4	
SSB	SSB	3102	57536	0.1	Yes.
Mitogen-activated protein kinase 8	MAPK8_tv1	9813	3972	3	
Nucleoside diphosphate kinase, mitochondrial	NME4	14721	3543	4	
Nuclear receptor-binding factor 2	NRBF2	20708	6088	4	
Phosducin-like protein 3	PDCL3	5688	12749	0.4	
Pyruvate kinase PKLR	PKLR	9696	3149	3	

POU domain class 2-associating factor 1	POU2AF1	8954	3692	2	
Proteasome activator complex subunit 3	PSME3	7824	2226	4	Yes.
Pyrroline-5-carboxylate reductase 1, mitochondrial	PYCR1	24271	6591	4	
Ribokinase	RBKS	11552	3167	4	
60S acidic ribosomal protein P1	RPLP1	15070	6012	3	Yes.
Protein TRK-fused gene protein	TFG	12939	4210	3	Yes.
Transketolase	TKT	9353	3523	3	Yes.
Tropomyosin alpha-3 chain	TPM3	10387	3591	3	
Zinc finger protein 496	ZNF496	1109	4676	0.2	
Zinc finger HIT domain-containing protein 3	ZNHIT3	8607	3878	2	

Unique targeted antigens in one of the two rheumatoid arthritis conditions. Proteins only targeted by IgG from either anti-CCP positive rheumatoid arthritis plasma or anti-CCP negative rheumatoid arthritis plasma are shown with fold-change and their average relative fluorescence unit intensity.
